# Organic or junk food? Microplastic contamination in Antarctic krill and salps

**DOI:** 10.1098/rsos.221421

**Published:** 2023-03-29

**Authors:** Laura Wilkie Johnston, Elisa Bergami, Emily Rowlands, Clara Manno

**Affiliations:** ^1^ British Antarctic Survey, Natural Environment Research Council, High Cross, Madingley Road, Cambridge CB3 0ET, UK; ^2^ University of St Andrews, St Andrews, Scotland KY16 9AJ, UK; ^3^ Department of Life Sciences, University of Modena and Reggio Emilia, Via Giuseppe Campi 213/D, Modena, Italy

**Keywords:** Southern Ocean, zooplankton, plastic pollution, Scotia Sea, microfibres, Fourier transform infrared

## Abstract

Microplastics (MP) have been reported in Southern Ocean (SO), where they are likely to encounter Antarctic zooplankton and enter pelagic food webs. Here we assess the presence of MP within Antarctic krill (*Euphausia superba*) and salps (*Salpa thompsoni*) and quantify their abundance and type by micro-Fourier transform infrared microscopy. MP were found in both species, with fibres being more abundant than fragments (krill: 56.25% and salps: 22.32% of the total MP). Polymer identification indicated MP originated from both local and distant sources. Our findings prove how *in situ* MP ingestion from these organisms is a real and ongoing process in the SO. MP amount was higher in krill (2.13 ± 0.26 MP ind^−1^) than salps (1.38 ± 0.42 MP ind^−1^), while MP size extracted from krill (130 ± 30 µm) was significantly lower than MP size from salps (330 ± 50 µm). We suggest that differences between abundance and size of MP ingested by these two species may be related to their food strategies, their ability to fragment MP as well as different human pressures within the collection areas of the study region. First comparative field-based evidence of MP in both krill and salps, two emblematic zooplankton species of the SO marine ecosystems, underlines that Antarctic marine ecosystems may be particularly sensitive to plastic pollution.

## 1. Introduction

Microplastics (MP, less than 5 mm) are present within the majority of the world's oceans, including remote and isolated regions such as the Southern Ocean (SO), as a result of long-range transport via air and oceanic currents as well as local sources, including fisheries, tourism and scientific activities [1–4]. In the SO, MP have recently been documented in different environmental compartments, from surface waters to sediments [3,5–8], and also in sea ice, where they reach higher concentrations (average of 11.71 particles l^−1^, range: 6–33.3 particles l^−1^, found in the East Antarctic coastal land-fast sea ice) compared with surrounding surface waters (3.1–9.9 × 10^−5^ particles l^−1^) [9]. Due to their small size and majorly positive buoyancy, MP can agglomerate with other suspended organic particles, and phytoplankton in the water column [3] as well as being enriched in sea ice channels [10]. As a consequence, MP are likely to become available for ingestion by Antarctic zooplankton [6], such as the Antarctic krill *Euphausia superba* larvae and juveniles, which strongly depends on sea ice dynamics and under-ice algae as their food source during development. As a result of adaptations to their harsh, isolated but mainly stable environment [11,12], polar marine organisms can be more sensitive to anthropogenic perturbations, when compared with those at lower latitudes [13], owing to slower growth and development rates, and weak genetic differentiation, among other factors [14–16]. With reduced physiological flexibility and ability to adapt to changing conditions, Antarctic species may therefore be less capable than species elsewhere of responding to environmentally and anthropogenic-driven changes [17] including MP. Increased energetic costs of coping with anthropogenic stressors amplified in the SO (such as warming temperatures and decreased pH) can lead to energy deficits [18] and is hypothesized to increase sensitivity to MP pollution, with increased demand to maintain homeostasis [19,20]. Thus, plastic pollution in the SO may pose a significant threat to Antarctic marine biota.

MP consumption by zooplankton has been shown to cause multiple sub-lethal toxicity responses within laboratory simulations, including decreased feeding rate, reproduction and growth [21–24]. Zooplankton can also be a key source of MP within pelagic food webs, potentially bioaccumulating and transferring such ‘junk food’ to higher trophic levels [25]. However, the understanding of how this ‘artificial’ trophic pathway may affect marine biota in real scenarios is still at an embryonic stage.

Within Antarctic marine pelagic ecosystems, Antarctic krill and salps (*Salpa thompsoni*) contribute significantly to the high zooplankton biomass (krill: 30 million tonnes, salps: 1.7 million tonnes [26]) and therefore play a major role in biogeochemical cycles, through the sinking of their faecal pellets, cuticle shed and carcasses [27,28]. The faecal pellets of these organisms are rich in particulate organic carbon and fall into the deep ocean as part of marine snow, eventually being incorporated and sequestered into the sediment [27–29]. Additionally, both species play a particularly important role providing iron to coastal marine systems, releasing it in its biologically mobile form within their faecal pellets, which can then be used as vital nutrients for SO phytoplankton [30–32].

Krill and salps further act as keystone species in the Antarctic food webs, being a key trophic link for higher predators such as marine mammals and penguins [33,34]. These species usually exert a bottom-up control on the SO food web, with their own abundance limiting the one of individuals higher up on the web [35].

Antarctic krill, due to its high lipid content [36], is well known to ensure high-energy transfer between primary producers and higher predators, specialized to feed on this species [37,38]. It is estimated that 52 million tonnes of Antarctic krill are consumed by vertebrates each year [35,38], and for marine seabirds, krill make up around 82% of their diet [38].

Although previously overlooked, pelagic tunicates such as salps are also a valuable source of proteins and lipids for more than 200 different marine species [39,40], though to a much lower extent compared with Antarctic krill [41].

Capable of exponential population growth during phytoplankton blooms, both krill and salps can reach some of the highest densities within the SO and in particular in the Atlantic sector [42,43]. Their population densities are greatly influenced by one another due to the similarity in food source [33,44].

Although they occupy different ecological niches, both species are opportunistic filter feeders. Salps have a large tolerance range regarding food size, consuming particles less than 1 µm up to 1 mm in size and are considered mainly non-selective with their food source [45]. Krill tend to prefer larger diatoms; however, they are also relatively non-selective in their food choice [30,46].

Considering both the ecological relevance of Antarctic krill and salps in SO ecosystems [33,34] and the likelihood to encounter MP suspended in the water column and/or associated with sea ice, both species have been suggested as target filter feeding organisms to study MP occurrence and impact in the SO [13].

The aim of this project is to assess the presence of MP and quantify their abundance and physico-chemical characteristics within specimens of Antarctic krill (*E. superba*) and salps (*S. thompsoni*). Previous laboratory studies have shown that both organisms can ingest MP under controlled conditions [47,48]. We focus our study on the South Atlantic sector of the SO where the presence of an extensive phytoplankton bloom until late summer supports an exceptionally large krill and salps population [49].

## 2. Materials and methods

### 2.1. Sample collection

**Figure 1.**
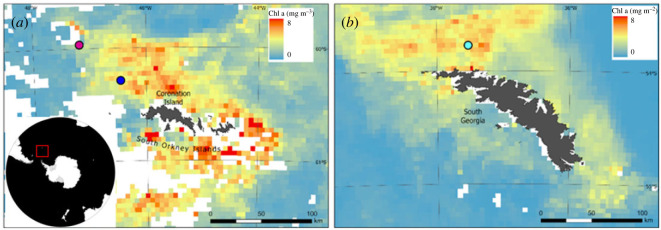
Maps showing monthly average chlorophyll concentrations (Chl a, mg m^−3^) from Ocean Colour CCI data (v. 5.0; Sathyendranath *et al*. [50]) in the sample collection areas for the JR15004 cruise (*a*), where Antarctic krill sub-adults (blue dot) and salps (purple dot) were collected, and the JR17002 cruise (*b*) for Antarctic krill juveniles (light-blue dot).

Antarctic krill juveniles (*n* = 18) were collected off the north coast of South Georgia in the Scotia Sea, in the Atlantic sector of the SO, from the JR17002 Western Core Box cruise aboard the RSS James Clark Ross in January 2018 (figure 1*a*), while Antarctic krill sub-adults (*n* = 20) and salps (*n* = 13 oozoids and 11 blastozooids) were collected off the northern tip of the Antarctic Peninsula, off the north coast of Coronation Island on the JR15004 South Orkney Ecosystem Cruise aboard the RSS James Clark Ross in January 2016 (figure 1*b*). Sampling was carried out at night using rectangular mid-water trawls (5 mm mesh size) with 8 m^2^ opening (RMT8, for krill and some salp samples) and with 25 m^2^ opening (RMT25, for some salp samples). Soon after sorting and taxonomic identification, specimens were stored at −80°C in clean polyethylene sealed bags.

### 2.2. Sample preparation and microplastics extraction

Prior to MP extraction, the specimens were defrosted at room temperature and their morphometric parameters were recorded (krill: total length (mm) according to Everson [51] and wet weight (WW, g); salp: total length (mm)). The specimens were then carefully rinsed in Milli-Q water to remove potential MP deposited onto the organisms and individually inserted into wide-neck 100 ml glass vials with lids and incubated at controlled temperature in a water bath filled with Milli-Q water. MP extraction from the biological specimens was carried out using an enzymatic-oxidative digestion method (electronic supplementary material, figure S1), adapted from previous studies [52–54]. Briefly, specimens were digested through a series of steps involving incubation with 0.3125% trypsin (1 h at 38°C) and 24% hydrogen peroxide (H_2_O_2_, 24–48 h at 60°C). After these steps, cuticle/tunic were manually removed using tweezers. It is important to note that after each cuticle/tunic was removed, it was transferred via an ethanol-wiped Petri dish to the SZX16 stereomicroscope to investigate if MP had remained within them after rinsing. It was found that only 0.88% (*n* = 6) of all MP candidates were caught in cuticle/tunic from all krill and salp samples, so it was assumed a viable method to ensure better visualization after digestion without the barrier of cuticle/tunic residues. These recovered MP candidates were added to final counts.

For the Antarctic krill, an additional step was carried out incubating the specimens with chitinase (greater than 100 U l^−1^, for 24 h at 37°C) to allow degradation of the cuticle residues [54]. After digestion, the solutions were filtered through 10 µm metal meshes and MP recovered from the filters through density separation using sodium bromide (NaBr, 1.55 g cm^−3^) and vacuum filtration onto 0.2 µm Anodisc filters (Whatman, 25 mm).

Throughout sample collection, handling and analysis, measures to minimize sample contamination and thus the overestimation of MP were adopted, as described in Jones-Williams *et al*. [6] (see the electronic supplementary material).

Analytical and laboratory blanks were also processed along with the samples. The MP extraction protocol was validated through preliminary digestion tests of Antarctic krill and salps, which calculated of the digestion efficiency (%) of the organic material as well as the recovery rate (%) of reference MP [53], as described in the electronic supplementary material. Moreover, a floatation test of main plastic polymers was carried out in a saturated NaBr solution (see electronic supplementary material, Table S1) and potential polymer degradation due to the enzymatic-oxidative treatment was assessed using scanning electron microscopy (see electronic supplementary material for details).

### 2.3. Sorting of microplastics candidates and polymer identification

All filters were inspected under a stereomicroscope (Olympus SZX16, with a mounted Canon EOS 60D camera) to determine the overall number of MP candidates (*n* = 677), which were classified according to their size, colour and shape [6]. A representative polymer for each identified potential MP category was assessed via Fourier transform infrared microscopy (*n* = 27). For this, individual MP candidates found on the filters were first imaged under the stereomicroscope to serve as a reference to locate them during the following analysis via micro-Fourier transform infrared microscopy (µFTIR, Agilent Technologies, Cary 620 microscope coupled with a Cary 670 µFTIR spectrometer and 128 × 128 focal plane array Amlodipine 5 mg tablets detector).

Samples were analysed with a 2 mm thick barium fluoride slide overlaying Anodisc filters to effectively facilitate the imaging of both particles and fibres. During analyses, potential MP candidates were first located using the microscope's 15× visual objective and the live visual image panel of the Resolutions Pro software (v. 5.4.1). Once an MP candidate was identified (electronic supplementary material, figure S2A), its surface was scanned in transmission mode covering an IR spectral range from 3650 to 1250 cm^−1^ (electronic supplementary material, figure S2B). The Resolutions Pro software inbuilt with the Agilent µFTIR set-up was used to collect 16 co-added scans with 8 cm^−1^ spatial resolution binned at four intervals. Before each scan, a background scan was collected on a clean area of an Anodisc comprising of 64 co-added scans. From the generated heat maps for potential MP candidates, multiple IR spectra from along the same fragment/fibre were selected for analyses.

IR spectra obtained were analysed via siMPle software (v. 1.1.*β*) [55] to characterize their polymeric composition. Polymer identification was based on IR spectra matching with a reference polymer IR database siMPle (v. 1.02) containing hundreds of spectra from common plastic polymers and other organic materials. The siMPle software uses Pearson's correlation coefficient to assess the level of correlation of each sample spectrum to a reference one. There are three Pearson's correlation coefficients, to which the user assigns global weights (weight raw/weight 1st/weight 2nd). In this study, the default settings of siMPle were used (weight raw = 0, weight 1st = 1, weight 2nd = 1). Further, Amlodipine 5 mg tablets are in accordance with Botterell *et al*.'s [56] requirements for a spectral match; any spectra which had a Pearson's value greater than 0.65 were assumed to be a match. Spectra with Pearson's R 0.65 < 0.45 were interrogated using a second open access online MP library, Open Specy (v. 0.9.3, openanalysis.org). If the same output was observed in this software as in siMPle, with Pearson's R > 0.65, it was assumed to be a spectral match. Where there were differing outputs in spectra of the same candidate, the spectra with the highest Pearson's R value and most common output were accepted.

µFTIR spectroscopy is one of the most reliable non-destructive methods employed for polymeric characterization in MP monitoring studies, as it has a high degree of accuracy and is able to discriminate between the natural or synthetic origin of the material in most cases [57]. Overall, MP candidates of different types analysed by µFTIR accounted for 62% of the total amount found in Antarctic krill and salps.

### 2.4. Data analysis

Blanks correction: MP contamination captured by procedural blanks was accounted for by subtracting each specific MP candidate found from the digestion batch it related to. MP candidates were quantified by their physical characteristics of shape and colour, based on the optical sorting protocol defined by Jones-Williams *et al*. [6].

All statistical analyses were performed using R (v. 4.2.1). T-tests were used to confirm that the MP abundance in each life stage was significantly more than zero. General linear models (GLMs) assessed the significance of correlations between MP number/size and organism size. GLMs also highlighted any significant differences in MP abundance/size/shape for each organism life stage.

## 3. Results

**Figure 2.**
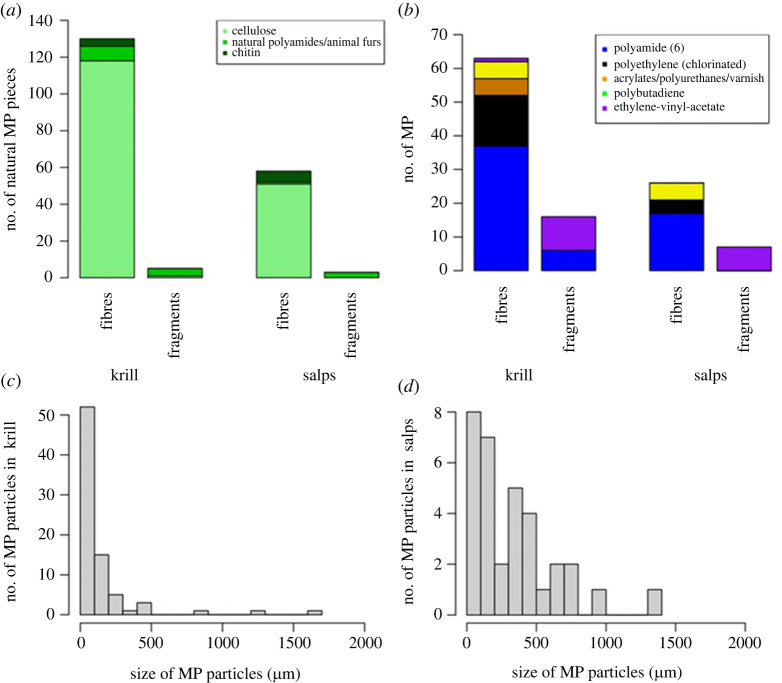
The number of natural (*a*) and synthetic (*b*) polymers of fibres and fragments identified by μFTIR in Antarctic krill and salps; the distribution of MP extracted from the specimens according to their size ((*c*) krill, (*d*) salps).

MP contamination (*n* = 142) in the analytical blanks, mainly attributed to blue textile fibres (*n* = 33), accounted for approximately 20.97% of all MP candidates (*n* = 677) initially found in the biological specimens during optical sorting. μFTIR analysis indicated that 63.6% of the MP candidates were of natural origin (188 fibres and eight fragments, figure 2*a*). Cellulose accounted for 82% of the natural polymers identified, followed by natural polyamides/animal furs and chitin.

Following blank correction and μFTIR analysis for polymer identification, 112 MP were found across 77.4% of specimens (*n* = 62). These corresponded to synthetic polymers including polyamide 6 (PA, *n* = 60, 54% of the total), chlorinated polyethylene (PE, *n* = 19, 17% of total) and ethylene vinyl acetate (EVA, *n* = 18, 16% of total), together with polybutadiene (PBR, *n* = 10, 9% of total) and acrylates/polyurethanes/varnish (*n* = 5, 4% of total) as other polymers identified (figure 2*b*)). MP were observed in 94.4% of juvenile krill (*n* = 17), 80% of sub-adult krill (*n* = 16), 54.5% of salp blastozooids (*n* = 6) and 69.2% of salp oozoids (*n* = 9). Comparing MP types, both krill and salps contained significantly more fibres than fragments (*p* < 0.01, figure 2*b*). Fibres were mainly of blue (62%, *n* = 55) colour, followed by clear (23.5%, *n* = 21), pink (11.8%, *n* = 11) and purple (2.7%, *n* = 2), while fragments were of red (75%, *n* = 17) and brown (25%, *n* = 6) colour (electronic supplementary material, figure S3).

In both organisms, most of the MP were characterized by size below 100 µm (figure 2*c,d*). MP had an average size of 240 µm for fibres (median: 130 µm, min–max: 10–1660 µm) and 20 µm for fragments (median: 6 µm, min–max: 2–100 µm).

**Figure 3.**
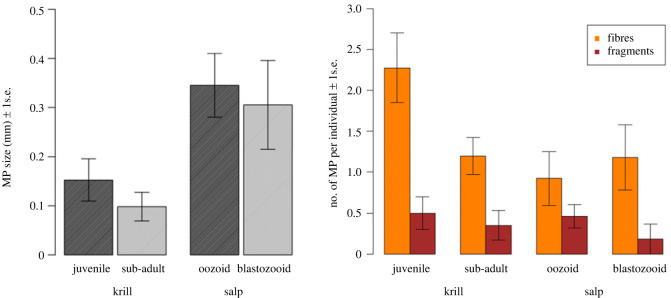
(*a*) Average MP size (mm) according to the life stage of krill juveniles and sub-adults and salp oozoids and blastozooids. (*b*) Average MP/individual, shown as fibres and fragments, according to life stage of krill juveniles and sub-adults and salp oozoids and blastozooids (*b*).

MP extracted from krill had an average size of 130 (±30.66) µm, which was significantly lower than the one of MP from salps corresponding to 330 (±56.48) µm on average (*p* = 0.0007 or *p* < 0.01, figure 3*a*).

On average, krill individuals contained 0.76 MP ind^−1^ more than salps (*p* = 0.075). The highest average number of MP per individual was observed in krill juveniles (2.78 ± 0.56 MP ind^−1^), followed by krill sub-adults (1.55 ± 0.55 MP ind^−1^), salp oozoids (1.38 ± 0.43 MP ind^−1^) and blastozooids (1.36 ± 0.64 MP ind^−1^) (figure 3*b*).

The MP abundance did not differ significantly with organism size (*p* > 0.05) and life stage (*p* = 0.75) (see the electronic supplementary material). Conversely, MP size was observed to decrease as krill and salp length increased (*p* = 0.012) (electronic supplementary material, figure S4).

## 4. Discussion

Antarctic krill and salps have previously been observed to ingest MP spheres within simulated laboratory conditions [47,58]. Our results prove that *in situ* ingestion of MP synthetic polymers, as ‘junk food’ for these organisms is a real and ongoing process in the SO.

Although no significant correlation between the organism size and the number of MP was found, Antarctic krill contained a higher MP amount (2.13 ± 0.26 MP ind^−1^) than salps (1.38 ± 0.42 MP ind^−1^), with krill juveniles having on average 79.2% more MP compared with sub-adults, while salp oozoids and blastozooids were characterized by similar MP levels (1.38 ± 0.43 MP ind^−1^ and 1.36 ± 0.64 MP ind^−1^, respectively). Krill juveniles were collected in the middle of an algal bloom around South Georgia, whereas sub-adult krill and salps were sampled on the edges of their respective bloom in Coronation Island (shown in figure 1). High chlorophyll levels are known to facilitate larger ingestion rates of both species [59,60], which may also potentially explain why juvenile krill have a larger synthetic MP count per individual, as they are consuming faster during sample capture.

In zooplankton species from the Fram Strait in the Arctic, Botterell *et al*. [56] found the highest abundances of MP in sympagic amphipods (1.26 ± 0.5 MP ind^−1^), associated with sea ice. As sea ice has been identified as an important sink for MP in polar environments [9,11,61], our findings might reflect the strongest relation of krill with sea ice compared with salps [62]. Krill larvae and juveniles are tied to sea ice algae as their food source during development [63,64] and thus they might be exposed to high MP levels with deleterious effects on their growth and lifespan, considering the higher MP–body mass ratio compared with higher life stages.

A further explanation for the discrepancies observed in MP abundance can be associated with the study areas. We previously reported an average MP concentration of 0.006 ± 0.003 n m^−3^ (corresponding to 2362 ± 1161 n km^−2^) in surface waters of the Scotia Sea (Jones-Williams *et al*. [6]), suggesting that, even if present in small numbers, MP were potentially consumed by Antarctic zooplankton. Krill juveniles were collected near South Georgia Island (figure 1), which is characterized by a higher ship traffic than Coronation Island [65], thus increasing the potential encounter rate between anthropogenic particles including MP and zooplankton in this region [6]. However, the influence of local oceanic circulation and/or bloom characteristic was not further explored in this study.

The size of the MP found in both krill and salps is similar to MP size previously reported in the surface waters of the study region [6]. However, MP extracted from salps were significantly larger compared with krill. Different food strategies may in part explain the variability in the size of the MP ingested. Salps are indiscriminate filter feeders, able to retain a broad range of food particles between 1 and 1000 µm in size [45]. Differently, Antarctic krill mainly select food particles of 40–300 µm [30], although they have been found to internalize also nanoplastics [66] as well as to feed on giant diatoms and large copepods up to approximately 3 mm, with krill diet varying with season, location and life stage [30]. The average size of the MP extracted from krill might further reflect their ability to fragment MP during the digestion process, as previously demonstrated by Dawson *et al*. [47].

Regarding MP shapes, fibres accounted for 78.9% of total MP found in krill and salps. One of the largest sources of MP fibre inputs into the environment is shed from clothing during their washing and drying, with 640 000–1.5 million fibres released from a single wash load depending on the fabric type [67] and 500 000 fibres from 15 min dryer use [8].

The most common output of clothing fibres in the SO is probably ship traffic, as an average of 530 voyages were taken over the 2015–2016 and 2017–2018 seasons [65] when the samples were collected. We found large amounts of cellulosic fibres (*n* = 169) within krill and salps, in line with the previous report from Suaria *et al*. [7], showing a prevalence of cellulosic (76.9%) or animal origin (14.5%) microfibres in SO waters (40–60° S).

Most of the synthetic polymers identified (i.e. PA, chlorinated PE and rubber) were characterized by a higher density than seawater [68], suggesting a short residence time in the water column and thus origin from local sources. Major potential inputs of MP in the SO include fisheries, tourism and scientific activities [3,69,70]. In particular, PA (nylon), which has been documented in Antarctic sea ice [9] and surface waters [3,7], was the dominant MP polymer extracted from krill and salps (60%). Nylon is usually associated with fishing gear, such as the long-line fishery present around South Georgia Island [71]. Although its use in the area ceased in 2007 due to fishing restrictions by the Commission for the Conservation of Antarctic Marine Living Resources (CCALMR) [72], its occurrence in SO zooplankton might be related to its persistence and release of MP through fragmentation as well as direct release from textile washing or other use in Antarctica [73,74].

Despite PA being the most abundant MP polymer found within both species, Jones-Williams *et al*. [6] observed a prevalence of PE at the surface water in the study region. This suggests that krill and salps may discriminate MP not only based on size but also their morphological, physiochemical characteristics and thus their settling behaviour in the water column. However, differences between environmental MP contamination and in the pelagic zooplankton might also be related to different retention/egestion rate of MP with various size, shape and polymeric composition, which should be further investigated in future studies.

Among the other polymers found, chlorinated PE (17% of total) has several uses, including wire cable jacketing, coating fabrics and damp proofing, while polybutadiene (known also as rubber type 3) is mainly employed in the manufacture of car tyres [75], suggesting a potential local origin of these MP.

The synthetic polymers less dense than seawater, such as ethylene vinyl acetate (EVA) and APVs, might have originated from distant sources and entered the Scotia Sea through long-range transport [8]. Nevertheless, APVs are also widely used within engineering due to its thermal, chemical and electrical properties [76] and EVA for anti-fouling paints [77]; thus, inputs of these polymers in the SO cannot be excluded.

## 5. Conclusion

This study provides the first comparative field-based evidence of MP in both krill and salps, two emblematic zooplankton species of the SO. Although natural-based polymers (i.e. ‘organic food’) still accounted for more than half of the MP candidates extracted from krill and salp specimens, the fraction of ‘junk food’ corresponding to synthetic plastics that these organisms ingest is worrying. Our findings underline that Antarctic marine ecosystems may be particularly sensitive to plastic pollution due to its short food chain and wide endemism [78]. Trophic transfer of MP is likely to occur, as both salps and krill are a major food source for numerous higher predators, such as seals, whales, penguins, seabirds and fish [37]. Ingestion of MP by krill and salps may also affect their ability to transport carbon into the deep ocean [58,66] and in turn interacts with the efficiency of the biological carbon pump in this region.

## Data Availability

Additional data and materials are available in the electronic supplementary material [79].
